# Effect of laying time on hatching results and some egg quality characteristics in Caucasian pheasants

**DOI:** 10.1016/j.psj.2025.105787

**Published:** 2025-09-03

**Authors:** Emre Arslan, Mahmut Şamil Şamlı, Merve Tok, Gülce Kırbaş, Özlem Karaman, Kemal Kırıkçı

**Affiliations:** a Department of Animal Science, Faculty of Veterinary Medicine, University of Selçuk, Konya, Türkiye; b Department of Animal Science, Faculty of Veterinary Medicine, University of Hatay Mustafa Kemal, Hatay, Türkiye

**Keywords:** Pheasant, Oviposition time, Hatchability, Chick quality, Egg characteristics

## Abstract

This study aimed to investigate the effects of oviposition time on egg quality, hatchability, and chick quality in Caucasian pheasants *(Phasianus colchicus*). A total of 40 female and 4 male pheasants were used in a controlled trial. Eggs were collected twice daily—once in the morning (10:00–11:00) and once in the afternoon (16:00–17:00)—over a 120-day period. Randomly selected 60 eggs were analyzed for internal and external quality characteristics, and incubated to assess hatchability and chick quality, the latter evaluated using the Tona scoring system.

The results showed that 66.82 % of eggs were laid in the afternoon, indicating a species-specific laying rhythm. While oviposition time did not significantly affect egg quality parameters such as shell thickness, albumen index, or Haugh units, it had a significant impact on hatchability and chick quality. Afternoon-collected eggs yielded higher hatchability rates and better chick quality scores.

Based on these findings, adding a third daily egg collection may reduce storage time and improve chick viability. Oviposition time is a manageable factor that can enhance reproductive efficiency, and adjusting collection schedules to natural laying rhythms offers a simple, low-cost way to improve hatchery results. Future studies should also consider environmental and nutritional factors.

## Introduction

The timing of oviposition in avian species has long been a subject of interest to researchers, due to the potential influence of this behavior on egg quality and reproductive success. While some species of bird exhibit highly synchronized laying behaviors, producing eggs at nearly the same hour each day, others display considerable variability in their oviposition times. For instance, the blue peafowl (*Pavo cristatus*) has been observed to typically deposit its eggs between 18:00 and 20:00 hours. It is therefore recommended that collections of eggs be made in the short period following this timeframe, in order to ensure optimal handling (Kırıkçı, 2012).

Comparative studies among bird species have shown a general trend in which smaller species lay earlier in the day, with shorter oviposition durations compared to larger-bodied species (McMaster et al., 2004). Funk (1934) found that eggs collected between 12:00 and 14:00 had higher hatchability than those collected at 09:00, despite differences in species. In wild pheasants, most eggs were laid between 10:00 and 15:00 (Klonglan et al., 1956). In commercial poultry farming, morning-laid eggs are often heavier and more numerous (Boz, 2011), although fertility and hatchability generally remain unaffected; however, chick hatch weight can be influenced by laying time. Later-laid eggs have been reported to be lighter but with improved shell quality (Onbaşılar and Avcılar, 2011), while yolk protein is higher in morning eggs and albumen protein and yolk lipid are greater in afternoon eggs (Tabib et al., 2021). Findings on internal quality traits such as Haugh unit (Eleroğlu, 2021; Altun et al., 2022) and shell weight ( Onbaşılar and Tabib, 2019) remain inconsistent, though some evidence suggests that later-laid eggs may exhibit higher hatchability (Abacı et al., 2020). Similar patterns have been observed in quails and partridges, where oviposition time affects egg production and quality but not fertility or hatchability (Söğüt and Mustafa, 2009; Çetin et al., 2008). In pheasants, egg quality is further shaped by shell colour (Kırıkçı et al., 2005) and breeder age, with advancing age associated with thinner shells, reduced yolk traits, and lower Haugh units alongside increases in albumen measures (Kuzniacka et al., 2005; Esen et al., 2010; Günlü et al., 2018). Overall, hatchability outcomes are influenced by fundamental egg traits such as weight, shape index, shell thickness, and the relative contributions of yolk, albumen, and shell to total egg mass (Kuzniacka et al., 2005; Tserveni-Goussi and Fortomaris, 2011; Galic et al., 2023). Likewise, chick quality—an essential factor for the efficiency of poultry production—is shaped by a complex interaction of genetic and environmental conditions, and can be improved through effective early selection of high-quality chicks (Okur and Türkoğlu, 2011; Cam et al., 2024).

Despite the growing interest in pheasant farming in Türkiye and worldwide, practical knowledge regarding egg quality and incubation performance, particularly in relation to oviposition time, remains limited. Few comprehensive studies have examined how laying time affects egg traits and hatchability, highlighting the need for controlled research in this field. Therefore, the aim of the present study was to determine the effects of oviposition time on egg quality, hatchability, and chick quality in Caucasian pheasants (*Phasianus colchicus*).

## Materials and methods

### Ethics committee approval

This study was approved by the Ethics Committee of Selçuk University, Faculty of Veterinary Medicine, Experimental Animals Production and Research Center (Approval No: 2024/048; Meeting Date: 28.03.2024).

### Animals and experimental site

The experimental material consisted of 40 female and 4 male Caucasian pheasants (*Phasianus colchicus*). The birds were obtained at 20 weeks of age from the Polonezköy Pheasant Breeding Farm, operated under the General Directorate of Nature Conservation and National Parks, Ministry of Agriculture and Forestry. The study was conducted at the Alternative Poultry Unit of the Research and Application Farm, Faculty of Veterinary Medicine, Selçuk University.

### Husbandry and management

The pheasants were housed in outdoor aviaries measuring 4.0 *m* × 5.0 *m* × 2.5 m, at a sex ratio of 1 male to 10 females per pen. To prevent cannibalism, all birds were fitted with anti-pecking devices. During the laying period, pheasants were fed a layer diet containing 18 % crude protein *ad libitum*, and fresh water was supplied continuously via automatic drinkers. Nest boxes and perches were installed within each aviary.

Natural daylight was used as the only light source until the onset of laying. Following the first oviposition, artificial lighting was incrementally increased by one hour per week until reaching a 16 hours light:8 hours dark photoperiod, which was maintained for the duration of the study.

### Oviposition time of eggs and experimental design

Eggs were collected twice daily: in the morning (10:00–11:00) and in the afternoon (16:00–17:00). Egg production was monitored over a period of (120 days) from April-July 2024.

Every 15 days, 10 eggs from each oviposition time (morning and afternoon) were selected in triplicate (*n* = 30 per group) for egg quality analysis.

### Egg quality measurements

Egg weight, shell weight, and shell membrane weight were measured using a precision digital scale accurate to 0.01 g. Egg length, width, shell thickness, and membrane thickness were measured using a digital caliper accurate to 0.01 mm. Egg quality traits were calculated using the following formulas (Haugh, 1937;Yannakopoulos and Tserveni-Gousi, 1986)

ShapeIndex(%)=(EggWidth/EggLength)×100

$$\text{Shell}\;\;\text{Surface}\;\;\text{Area}\left(cm^{2}\right)=3.9782\times \;\text{Egg}\;\;{\text{Weight}}^{\wedge }0.75056$$

ShellPercentage(%)=(ShellWeight/EggWeight)×100

AlbumenIndex=[AlbumenHeight/((AlbumenLength+AlbumenWidth)/2)]×100

AlbumenWeight(g)=EggWeight−(YolkWeight+ShellWeight)

AlbumenPercentage(%)=(AlbumenWeight/EggWeight)×100

YolkIndex=(YolkHeight/YolkDiameter)×100

YolkPercentage(%)=(YolkWeight/EggWeight)×100

YolktoAlbumenRatio(%)=(YolkWeight/AlbumenWeight)×100

$$\text{Haugh}\;\;\text{Unit}=100\times \log[\text{Albumen}\text{Height}-\left(1.7\times \text{Egg}{\text{Weight}}^{\wedge }0.37\right)+7.57]\;\left(\text{Haugh},\;1937\right)$$

### Incubation and chick quality evaluation

Total of eggs were 1668 that were stored and incubated. Egg number of morning group was 555 and afternoon group was 1113. Formaldehyde and potassium permanganate, 14 ml: 7 g for 10 min., were used to fumigate eggs. Eggs were turned at 45° angle from vertical planes one time per hour. Egg-holding capacity of the setter was 10000 eggs. On the pre-incubation, the eggs were numbered for each group identification. Each group was randomly allocated within the setter.

Eggs were stored at 15°C and 75 % relative humidity for 7 days in a storage setter (HD-960L-3in1), then pre-warmed at room temperature (∼18 hours) before incubation. Eggs were fumigated prior to setting and incubated in a forced-draft setter at 37.7°C and 60 % humidity for 23 days. On day 24, they were transferred to a hatcher (Çimuka T1600 C) operating at 37.2°C and 85 % humidity.

Chick quality was assessed on the day of hatch using the scoring system proposed by Tona et al. (2003), based on the following parameters:

Activity: Ability to right themselves (Good = 6; Poor = 0)
Appearance: Feather dryness and cleanliness (Dry & Clean = 10; Wet = 8; Wet & Dirty = 0)
Yolk Absorption: Abdominal firmness and contour (Normal = 12; Wide/Firm = 0)
Eyes: Brightness and openness (Bright = 16; Dull = 8; Closed = 0)
Legs: Posture and inflammation (Normal = 16; 1 Leg Infected = 8; 2 Legs Infected = 0)
Navel: Closure and color (Normal = 12; Open = 6; Open & Pale = 0)
Membrane: Residual membrane size (None = 12; Small = 8; Large = 4; Very Large = 0)
Unabsorbed Yolk: Yolk mass size (Small = 16; Medium = 12; Large = 8; Very Large = 4; Extreme = 0)

Hatchability was determined on day 28 by calculating the number of unhatched eggs. Incubation performance was evaluated based on hatch averages obtained at 48, 52, and 56 weeks of age:

Hatchability(%)=(NumberofHatchedChicks/NumberofEggsSet)×100

### Statistical analysis

The normality of data distributions was assessed using the Kolmogorov–Smirnov test. Differences between morning and afternoon groups in terms of egg quality traits and egg weight loss were evaluated using the independent samples *t*-test. The association between oviposition time and hatchability was analyzed using the Chi-square test. The significance level of *P* < 0.05 was considered statistically significant. Analyses were performed using SPSS version 25.0.

Chick quality scores based on Tona et al. (2003) were analyzed on the day of hatch. Additionally, Python software was used to perform Chi-square analyses on categorical chick quality data, with a 95 % confidence interval.

## Results

The egg production rates of Caucasian pheasants according to oviposition time are presented in Table 1. As shown in the table, the oviposition time of eggs had a significant effect on egg production and laying efficiency in pheasants (*P* < 0.001). On average, 66.82 % of the daily collected eggs were laid between 16:00 and 17:00.

**Table 1:** Egg production of Caucasian pheasants according to laying time (*x*±Sx).

Oviposition time	Egg yield (%)	Yield (%)
10:00-11:00	22.48 ± 1.28	33.18 ± 1.78
16:00-17:00	44.92 ± 1.57	66.82 ± 1.78
Total	67.40 ± 2.02	100
P	***	***

***; (*P* < 0.001).

The effect of oviposition time on external egg quality traits is presented in Table 2.

**Table 2:** The effect of oviposition time on egg external quality characteristics in Caucasian pheasants (*x* ± Sx).

	10:00-11:00		16:00-17:00	General	P value
Shape Index	81.94 ± 0.52	81.46 ± 0.59		81.70 ± 0.39	NS
Egg weight (g)	31.80 ± 0.40	32.08 ± 0.37		31.94 ± 0.27	NS
Albumen Percentage (%)	30.74 ± 0.41	29.00 ± 1.32		29.92 ± 0.66	NS
Yolk Percentage (%)	58.77 ± 0.61	60.53 ± 1.39		59.60 ± 0.73	NS
Shell Percentage (%)	10.49 ± 0.51	10.46 ± 0.20		10.48 ± 0.28	NS

NS: not significant.

Table 3 presents the internal egg quality traits in relation to the time of collection. No statistically significant differences were observed in any of the internal quality parameters based on oviposition time of egg.

**Table 3:** Effect of oviposition time on egg internal quality characteristics in Caucasian pheasants (*x* ± Sx).

	10:00-11:00	16:00-17:00	General	P value
Albumen Index	2.62 ± 0.11	2.63 ± 0.08	2.62 ± 0.07	NS
Yolk Index	36.32 ± 0.76	36.21 ± 0.81	36.27 ± 0.55	NS
Haugh Unit	85.62 ± 1.15	85.55 ± 1.10	85.58 ± 0.79	NS

NS: not significant.

The effect of oviposition time on hatchability traits is given in Table 4. According to the incubation results, eggs collected in the afternoon exhibited significantly higher hatchability rates (*P* < 0.001). However, no significant differences were observed in fertility rates or embryonic mortality between oviposition times.

**Table 4:** The effect of oviposition time on hatching characteristics in Caucasian pheasants.

Features	10:00-11:00	16:00-17:00	P value
Hatchability (%)	64.71^a^	66.28^b^	***
Fertility rate (%)	89.68	89.59	NS
Hatchability rate of fertile eggs (%)	73.91	72.22	NS

(***:*P* < 0.001), NS: not significant.

The impact of laying time on chick quality is summarized in Table 5. The results indicate that both the proportion of high-quality chicks and the mean chick quality score were significantly influenced by the time of oviposition (*P* < 0.001). Specifically, chicks hatched from eggs collected in the afternoon exhibited superior quality scores compared to those from morning-laid eggs.

**Table 5:** Effect of laying time on chick quality in Caucasian pheasants.

Chick Quality Parameters	Oviposition time	
	10:00-11:00	16:00-17:00
Quality Chick Rate (%)	44,00^a^	77,00^b^
Average Quality Score of Chicks	96,68 ± 0,34^a^	98,62 ± 0,29^b^
P value	***	***

a,b : Differences between values with different letters in the same row are significant (***:*P* < 0.001).

## Discussion

### Egg production

The egg production rates of Caucasian pheasants at different oviposition times are presented in Table 1. It was found that the majority of eggs were laid in the afternoon (*P* < 0.001). Specifically, 66.82 % of daily eggs were collected between 16:00 and 17:00, while only 33.18 % were collected between 10:00 and 11:00. The overall daily egg production rate was calculated as 67.40 %, with 44.92 % of eggs collected during the afternoon period (*P* < 0.001).

These findings suggest that the oviposition rhythm of farmed Caucasian pheasants differs from that observed in species such as songbirds (McMaster et al., 2004), gadwalls (Oring, 1969), laying hens (Boz, 2011), and partridges (Çetin et al., 2008). However, the results are consistent with the observations of Klonglan et al. (1956) in wild pheasants. Moreover, similar to Caucasian pheasants, quails (Altan et al., 1995; Söğüt and Sarı, 2009) and peafowls (Kırıkçı, 2012) have been reported to predominantly lay eggs in the afternoon. In particular, Altan et al. (1995) and Baylan et al. (1998) reported that approximately 69.6 % and 67.89 % of daily egg production in quails occurred during the afternoon, respectively. These patterns suggest a species-specific tendency for egg production to peak at particular times of day in several poultry species.

### Egg quality traits

Optimizing the timing is economic significance, as it can influence overall egg quality (Kavtarashvili et al., 2019). In the present study, no statistically significant differences were found between morning and afternoon groups in terms of either internal or external egg quality traits in Caucasian pheasants (Table 2, Table 3).

Contrary to these findings, Eleroğlu (2021) reported significant effects of oviposition time on shape index, surface area, and egg volume in laying hens (*P* < 0.01). Studies on hens have also shown that egg weight varies with laying time (Patterson, 1997; Tůmová et al., 2009, 2017; Krawczyk et al., 2023). However, Çetin et al. (2008) found no effect of oviposition time on internal egg quality in partridges, which is consistent with the current findings in pheasants.

Regarding external traits, no significant effect of oviposition time was observed on shell percentage or shell surface area. Nevertheless, other researchers have reported species-specific effects of laying time on shell quality—for instance, Çetin et al. (2008) in partridges (*P* < 0.05) and Tůmová and Ebeid (2005) in hens (*P* ≤ 0.001). Such differences likely reflect interspecies physiological variation.

Tůmová and Ebeid (2005) also reported that oviposition time did not significantly affect the albumen ratio or yolk index, supporting our findings. However, they did observe significant effects on egg weight (*P* < 0.01), shape index (*P* < 0.01), albumen index (*P* < 0.05), and yolk percentage (*P* < 0.01), indicating that certain quality parameters are species- or breed-dependent.

In terms of albumen quality, no significant differences in Haugh Unit values were observed between eggs collected at different times of day, corroborating the results of Tůmová and Ebeid (2005), Altun et al. (2022), and Krawczyk et al. (2023).

### Incubation traits

Hatchability outcomes in pheasants are known to be influenced by environmental factors such as egg storage duration (Demirel and Kırıkçı, 2009), breeding systems (Kırıkçı et al., 2003), and lighting management (Tepeli et al., 1999). However, to the best of our knowledge, no prior studies have examined the direct effect of oviposition time on incubation traits in pheasants.

As shown in Table 4, oviposition time significantly affected hatchability, with eggs collected in the afternoon yielding higher hatchability (*P* < 0.001). No significant effects were observed on fertility or hatch rates. These findings are corroborated by research of Zakaria et al. (2009), who reported that oviposition time had no effect on hatching results and early or late embryonic mortality in hens.

### Chick quality

Oviposition time is a critical determinant of both egg quality (Tůmová and Ebeid, 2005; Hrnčár et al., 2013;Tumová et al., 2014 ; Shaker et al., 2019) and chick quality (Kamanlı and Durmuş, 2014). Numerous studies have indicated that afternoon-laid eggs exhibit improved shell quality (Pavlovski et al., 2000; Johnston and Gous, 2007), which can directly impact chick viability.

As shown in Table 5, chick quality scores were significantly higher in birds hatched from eggs collected in the afternoon (*P* < 0.001). One possible explanation is that morning-laid eggs remain in the nest for approximately 17 hours prior to collection, while afternoon-laid eggs are exposed for only about 5 hours. Prolonged nest exposure may negatively affect egg microclimate, increasing the risk of contamination or dehydration, thereby compromising chick quality.

Zakaria et al. (2009) also observed that although oviposition time did not affect fertility or hatchability directly, it did significantly influence egg weight and weight loss during storage, lending further support to our findings. Since egg weight plays a vital role in chick development, and oviposition time is known to influence egg weight (Tůmová and Ebeid, 2005; Tůmová et al., 2007, 2009; Egbeyale et al., 2013), the variation in chick quality may reflect cumulative effects of both weight and storage-induced changes.

Moreover, increased weight loss during storage in morning-collected eggs could impair embryonic development, leading to weaker or lower-quality chicks. Therefore, managing the timing of egg collection emerges as a critical factor in optimizing chick viability and overall production outcomes.

### Conclusions

This study demonstrates that oviposition time is a key factor influencing reproductive outcomes in Caucasian pheasants. While egg quality traits, fertility, and hatch rates were unaffected, afternoon-collected eggs showed higher hatchability and better chick quality. These findings emphasize that oviposition time, often overlooked in game bird production, can serve as a practical management variable to improve efficiency. Adjusting egg collection schedules to better align with laying patterns may represent a simple yet effective strategy for enhancing hatchery performance. Future research should further investigate how oviposition timing interacts with environmental and nutritional factors to support precision management in pheasant breeding systems.

## Disclosure statement

No potential conflict of interest was reported by the author(s).

## Funding

The study received financial support from Scientific Research Projects Coordination Unit, Selçuk University [Project Number: 24401074].

## CRediT authorship contribution statement

**Emre Arslan:** Conceptualization, Data curation, Formal analysis, Funding acquisition, Investigation, Methodology, Project administration, Resources, Software, Supervision, Visualization, Writing – original draft, Writing – review & editing. **Mahmut Şamil Şamlı:** Data curation, Resources, Software, Visualization, Writing – review & editing. **Merve Tok:** Data curation, Resources, Writing – review & editing. **Gülce Kırbaş:** Data curation, Resources, Writing – review & editing. **Özlem Karaman:** Data curation. **Kemal Kırıkçı:** Conceptualization, Investigation, Project administration, Supervision, Validation, Visualization, Writing – review & editing.

## Disclosures

The authors declare that they have no known competing financial interests or personal relationships that could have appeared to influence the work reported in this paper.

## Appendix Supplementary materials

Image, application 1
