# Supplementary material for: Effect of laying time on hatching results and some egg quality characteristics in Caucasian pheasants
Source: Poult Sci. 2025 Sep 3;104(11):105787. doi: 10.1016/j.psj.2025.105787 (PMC12683108; doi:10.1016/j.psj.2025.105787)
Supplement: Supplementary file 1 [file mmc1.pdf]

T.C.
SELÇUK ÜNİVERSİTESİ
VETERİNER FAKÜLTESİ DENEY HAYVANLARI ÜRETİM VE ARAŞTIRMA MERKEZİ ETİK KURULU (SÜVDAMEK) KARARLARI

Toplantı Tarihi	28.03.2024	Toplantı Sayısı	2024/03	Karar Sayısı	2024/048

S.Ü. Veteriner Fakültesi Öğretim Üyesi Dr. Öğr. Üyesi Emre ARSLAN tarafından sunulan ve Kemal KIRIKÇI, M. Şamil ŞAMLI, Özlem KARAMAN, Merve TOK ve Gülce KIRBAŞ'ın araştırmacı olarak yer aldığı **"Kafkas Sülünlerinde Yumurtlama Zamanının Kuluçka Sonuçları İle Bazı Yumurta Kalitesi Özelliklerine Etkisi"** başlıklı Araştırma Projesi başvurusu değerlendirilmiştir.

Başvuruda, Selçuk Üniversitesi Veteriner Fakültesi Deney Hayvanları Üretim ve Araştırma Merkezi Etik Kurulu (SÜVDAMEK) Yönergesi ilkelerine uyulduğuna, projenin araştırma etiği açısından "**Uygun olduğuna**" oy birliği ile karar verilmiştir.

Prof. Dr. Özgür ÖZDEMİR<br>Başkan	Prof. Dr. İbrahim AYDIN<br>Başkan Yardımcısı
Prof. Dr. Ayşe ER<br>Üye	Prof. Dr. Mustafa Selçuk ALATAŞ<br>Hayvan Refahı Birimi Üyesi

Prof. Dr. Nermin IŞIK USLU<br>Üye	Doç. Dr. Hasan ALKAN<br>Üye	Doç. Dr. Yusuf BİÇER<br>Raportör Üye
Vet. Hek. Dr. M. Sedat ARSLAN<br>Üye	Muhan ÜLGEN<br>Konya Doğayı ve Hayvanları Koruma Derneği Üyesi<br>(Katılmadı)	Sabri YALICI<br>Sivil Üye<br>(Katılmadı)
